# Mycobacterial Aminoglycoside Acetyltransferases: A Little of Drug Resistance, and a Lot of Other Roles

**DOI:** 10.3389/fmicb.2019.00046

**Published:** 2019-01-30

**Authors:** Fernando Sanz-García, Ernesto Anoz-Carbonell, Esther Pérez-Herrán, Carlos Martín, Ainhoa Lucía, Liliana Rodrigues, José A. Aínsa

**Affiliations:** ^1^Departamento de Microbiología, Facultad de Medicina – Instituto Universitario de Investigación de Biocomputación y Física de Sistemas Complejos, Instituto de Investigación Sanitaria Aragón, Universidad de Zaragoza, Zaragoza, Spain; ^2^Departamento de Bioquímica y Biología Molecular y Celular, Facultad de Ciencias – Instituto Universitario de Investigación de Biocomputación y Física de Sistemas Complejos, Universidad de Zaragoza, Zaragoza, Spain; ^3^Centro de Investigación Biomédica en Red Enfermedades Respiratorias, Instituto de Salud Carlos III, Madrid, Spain; ^4^Fundación Agencia Aragonesa para la Investigación y el Desarrollo, Zaragoza, Spain

**Keywords:** mycobacteria, aminoglycoside antibiotics, aminoglycoside acetyltransferase, drug target, pathogenicity, aminoglycoside resistance

## Abstract

Aminoglycoside acetyltransferases are important determinants of resistance to aminoglycoside antibiotics in most bacterial genera. In mycobacteria, however, aminoglycoside acetyltransferases contribute only partially to aminoglycoside susceptibility since they are related with low level resistance to these antibiotics (while high level aminoglycoside resistance is due to mutations in the ribosome). Instead, aminoglycoside acetyltransferases contribute to other bacterial functions, and this can explain its widespread presence along species of genus *Mycobacterium*. This review is focused on two mycobacterial aminoglycoside acetyltransferase enzymes. First, the aminoglycoside 2′-*N*-acetyltransferase [AAC(2′)], which was identified as a determinant of weak aminoglycoside resistance in *M. fortuitum*, and later found to be widespread in most mycobacterial species; AAC(2′) enzymes have been associated with resistance to cell wall degradative enzymes, and bactericidal mode of action of aminoglycosides. Second, the Eis aminoglycoside acetyltransferase, which was identified originally as a virulence determinant in *M. tuberculosis* (enhanced intracellular survival); Eis protein in fact controls production of pro-inflammatory cytokines and other pathways. The relation of Eis with aminoglycoside susceptibility was found after the years, and reaches clinical significance only in *M. tuberculosis* isolates resistant to the second-line drug kanamycin. Given the role of AAC(2′) and Eis proteins in mycobacterial biology, inhibitory molecules have been identified, more abundantly in case of Eis. In conclusion, AAC(2′) and Eis have evolved from a marginal role as potential drug resistance mechanisms into a promising future as drug targets.

## Bacterial Resistance to Aminoglycoside Antibiotics

Aminoglycoside (AG) antibiotics (Box 1) have not been an exception to the fact that after their introduction in clinical practice, resistance has been recorded (Waglechner and Wright, 2017). In fact, the three classes of aminoglycoside-modifying enzymes, aminoglycoside acetyltransferases (AACs), aminoglycoside phosphotransferases (APHs), and aminoglycoside nucleotidyltransferases (ANTs), have been widely detected in most pathogenic bacteria as a major determinant of resistance; in these bacteria the presence of aminoglycoside-modifying enzymes correlated with patterns of AG susceptibility (Smith and Baker, 2002). The modified AG (either by acetylation, phosphorylation or nucleotidylation) fails to inhibit their bacterial target, the 30S ribosomal subunit (Smith and Baker, 2002). Most genes encoding aminoglycoside-modifying enzymes are plasmid-located (indicative of a potential acquisition by horizontal gene transfer processes) and confer the bacterial hosts with high levels of AG resistance (Davies and Wright, 1997).

**Box 1.** Aminoglycosides: origins, structure, mode of action, resistance and clinical use.

Most aminoglycoside antibiotics are produced by bacterial species of the genus *Streptomyces*, such as the antitubercular streptomycin that is produced by *S. griseus* being the first antibiotic identified from bacteria. Other genera producing aminoglycosides are *Micromonospora* and *Bacillus*. Many semi-synthetic aminoglycosides, such as amikacin, have also been produced (Mingeot-Leclercq et al., 1999; Magnet and Blanchard, 2005; Shi et al., 2013).

Structurally, aminoglycosides are formed by an aminocyclitol (commonly, 2-deoxystreptamine) with additional amino sugars bound by glycosidic bonds. There are two large families of 2-deoxystreptamine aminoglycosides, those carrying substitutions at positions 4 and 5 of the 2-deoxystreptamine ring (including neomycin, paromomycin, lividomycin, ribostamycin and butirosin) and those being substituted at positions 4 and 6 of the 2-deoxystreptamine (including kanamycin, amikacin, tobramycin, dibekacin, arbekacin, gentamicin, isepamicin, sisomicin, and netilmicin). The carbon atoms in the sugar bound to position 4 of the 2-deoxystreptamine ring are named with primed numbers (’), and those in the sugar bound to positions 5 or 6 of the 2-deoxystreptamine ring are named with double-primed numbers (”). Other aminoglycosides contain aminocyclitols distinct to 2-deoxystreptamine (this is the case of streptomycin or apramycin) or are formed by fused amino sugar rings (i.e., spectinomycin) (Magnet and Blanchard, 2005; Shi et al., 2013). The following figure shows the structure of kanamycin A, an example of 4,6 di-substituted 2-deoxystreptamine aminoglycoside antibiotic.

**Figure:**
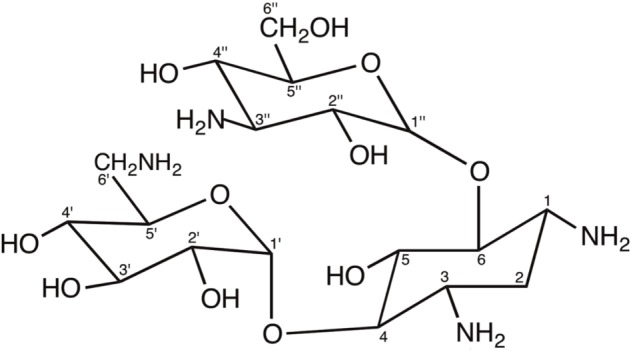

The bacterial target of aminoglycosides is the 30S small ribosomal subunit, and their global effect is the interference with protein synthesis. In bacterial cells, translation is initiated when the 30S ribosomal subunit binds the Shine-Dalgarno sequence (because of sequence complementarity between the Shine-Dalgarno sequence and the 16S rRNA molecule of the 30S ribosomal subunit), which is normally present in the 5′ untranslated region of mRNAs. Next, initiation factors and fMet-tRNA will join the complex that finally will recruit the 50S ribosomal subunit in order to start translation. Aminoglycoside binding to the 30S subunit does not affect the binding of mRNA and the large 50S subunit, so translation can proceed. However, aminoglycosides differ in their binding site at the 30S subunit, hence affecting the protein production at different levels. Whereas spectinomycin blocks translocation (hence being bacteriostatic), streptomycin and most 2-deoxystreptamine aminoglycosides lock the ribosome in a conformation that is prone to introducing erroneous aminoacyl-tRNAs. The accumulation of aberrant proteins in the bacteria results in cell death (Davies and Wright, 1997; Magnet and Blanchard, 2005; Shell et al., 2015).

Resistance to aminoglycosides may be due to several mechanisms (Davies and Wright, 1997; Magnet and Blanchard, 2005). Reduced uptake (which can be a consequence of alterations in the composition of bacterial membrane or to metabolic conditions like anaerobiosis) or the action of efflux pumps can lead to limited intracellular concentration of aminoglycosides, hence causing resistance. Mutations in 16S ribosomal RNA or certain ribosomal proteins such as S12 (encoded by *rpsL* gene) lead to aminoglycoside resistance through target modification; this is also achieved after the action of methyltransferases, which introduce methyl groups in guanine or adenine nucleotides of 16S ribosomal RNA. The presence of aminoglycoside-modifying enzymes is, however, the most prevalent mechanism of aminoglycoside resistance; there are three types of aminoglycoside-modifying enzymes: aminoglycoside *N*-acetyltransferases (AAC), *O*-phosphotransferases (APH), and *O*-nucleotidyltransferases (ANT). Aminoglycoside-modifying enzymes are named by using the aforementioned abbreviations, followed by a number in brackets indicating the site of modification in the aminoglycoside molecule (as explained above), a Roman numeral related with substrate profile, and a lower-case letter for differentiating isoenzymes, i.e., AAC(2′)-Ib.

Clinically, due to their oto- and nephrotoxicity and the rising prevalence of resistance, aminoglycosides are commonly reserved as a second line of treatment of serious infections. Due to their low absorption when given orally, aminoglycosides need to be administered through injections. Streptomycin was a first-line drug in the treatment of tuberculosis, and in our days, kanamycin and amikacin are listed as second line drugs against this disease. Spectinomycin is used against *Neisseria gonorrhoeae* infections (Suay-Garcia and Perez-Gracia, 2018). *Pseudomonas aeruginosa* infections in cystic fibrosis patients, septicemia, endocarditis and several other infections caused by non-tuberculous mycobacteria, Gram-positive or Gram-negative bacteria can be treated efficiently by using aminoglycosides, either alone or in combinations with other antibacterials such as the beta-lactam antibiotics (Mingeot-Leclercq et al., 1999; Magnet and Blanchard, 2005; Bassetti et al., 2018).

In mycobacteria, however, resistance to AGs resulted mainly from mutations of the ribosome components that prevent the drugs from inhibiting its function (Jugheli et al., 2009; Zhang and Yew, 2009; Shcherbakov et al., 2010). This is due to the fact that most mycobacterial species have either one (like *Mycobacterium. tuberculosis*) or two (like *Mycobacterium fortuitum*) ribosomal operons, hence making dominant those mutations acquired in their components (Magnet and Blanchard, 2005; Shi et al., 2013). The presence of aminoglycoside-modifying enzymes, mostly AAC, in mycobacterial species has been reported over the years (as detailed in the following sections), and the role of such AACs has been explored, originally for their contribution to AG resistance, and more recently for their role in other bacterial processes, which has resulted in the interest of developing inhibitors of these enzymes (Jana and Deb, 2005; Labby and Garneau-Tsodikova, 2013). In this review, we will summarize major findings on two mycobacterial AACs, the AAC(2′)-I and Eis enzymes, that have resulted in a Copernican turn for AACs in mycobacteria, from being putative drug resistance mechanisms, to reach the status of novel drug targets.

## Aacs in Non-Tuberculous Mycobacteria

The first detection of AACs in mycobacteria (Hull et al., 1984) was reported in a group of *M. fortuitum* isolates, an opportunistic fast-growing mycobacteria. Biochemical assays of crude extracts from *M. fortuitum* strains revealed the presence of AAC activity, strongly acetylating gentamicin and kanamycin A, along with other AGs. This substrate profile was consistent with that of AAC(3) enzymes that had been previously described in *Pseudomonas* and *Enterobacteriaceae* (Angelatou et al., 1982), although confirmation at the genetic or molecular levels were not done at that time. Surprisingly, the AG susceptibility profile of *M. fortuitum* could not be correlated with the activity of AACs, indicating that in this species AACs were not the major responsible for AG resistance; it was neither correlated with the presence of plasmids, hence suggesting a chromosomal location (Hull et al., 1984). In fact, the frequency of resistant mutants to kanamycin and amikacin in *M. fortuitum* and the related species *Mycobacterium chelonae* ranged between 10^-4^ and 10^-7^ (Wallace et al., 1985). This relatively high frequency of mutations, along with the fact that AAC activity was detected at similar levels between susceptible and resistant strains, led the authors to suggest that ribosome alterations were the main factor responsible of AG resistance in these species (Wallace et al., 1985). In another study (Udou et al., 1986), altered transport or permeability of AGs was identified as a contributor to AG resistance in *M. fortuitum*, since ribosomes from a clinical isolate were inhibited by one tenth of the MIC of AGs: for example, the MIC of kanamycin for *M. fortuitum* was 50 μg/ml, and in cell-free systems, 5 μg/ml of kanamycin reduced the activity of ribosomes to 13% in comparison with drug-free controls; similar results were obtained when using gentamicin or paromomycin (Udou et al., 1986).

The biochemical analysis of crude extracts from other non-tuberculous mycobacteria such as *Mycobacterium smegmatis, Mycobacterium phlei, Mycobacterium vaccae*, and *Mycobacterium kansasii*, from both clinical and environmental origins, revealed similar characteristics to those found in *M. fortuitum*: crude extracts from all strains contained AAC enzymatic activity, but no correlation with AG susceptibility profile could be established (Udou et al., 1987; Ho et al., 2000). In other mycobacterial species such as *Mycobacterium avium* and *Mycobacterium intracellulare*, however, AAC activity could not be detected (Ho et al., 2000). In a recent study, using cell-free translation assays, ribosomes of *Mycobacterium abscessus* and *M. smegmatis* were inhibited by both AGs having a 2′-amino group (tobramycin, dibekacin, and kanamycin B) and by those having a 2′-hydroxyl group (amikacin, and kanamycin A). However, in *M. abscessus*, those AGs having a 2′-amino group (tobramycin, dibekacin, and kanamycin B) were less efficient in killing the cells and inhibiting its ribosomes, which is consistent with the presence of a highly active AAC(2′) activity in this species. These findings suggested that in *M. abscessus*, AACs could somewhat mitigate the bactericidal effect of its AGs substrates (Maurer et al., 2015).

Interestingly, when characterizing the enzymatic activity of crude extracts, early reports detected that substrates such as amino sugars, malonyl-CoA, propionyl-CoA or butyryl-CoA inhibited the enzymatic activity of AACs from mycobacterial species. Such effect of non-acetyl CoA donors had never been described for the AAC(3) enzymes from other bacteria (Udou et al., 1987). Altogether, these findings strongly suggested for the very first time that, in mycobacteria, AACs could have important metabolic functions, and their contribution to AG resistance could be marginal (Udou et al., 1989).

## Aac(2′), a New Enzyme Comes Into Scene

In *Providencia stuartii*, a Gram-negative species, phylogenetically distant from mycobacteria, a novel class of AAC was identified as AAC(2′), because of its acetylating activity against gentamicin and lack of enzymatic activity against kanamycin A. The gene encoding AAC(2′)-Ia was cloned from *P. stuartii* (Rather et al., 1993) and found to be present in the chromosome of all isolates of this bacteria. In *P. stuartii*, the expression of the *aac(2′)-Ia* gene was controlled by several transcriptional regulators (Macinga and Rather, 1999), suggesting that this enzyme could play an important role, beyond its contribution to drug resistance (Franklin and Clarke, 2001). In fact, AAC(2′)-Ia contributes to *O*-acetylation of peptidoglycan affecting cell morphology and the expression of autolysins, and can use acetylated peptidoglycan precursors as donors of acetyl groups, another indication about the role of this enzyme beyond resistance (Payie et al., 1995, 1996; Clarke et al., 1996; Payie and Clarke, 1997).

### AAC(2′) in Mycobacteria

Given the high AG acetylating activity against gentamicin, tobramycin, netilmicin and its derivatives 2′-*N*-ethyl- and 6′-*N*-ethyl-netilmicin, kanamycin A and kanamycin B found in *M. fortuitum* (Hull et al., 1984; Ainsa et al., 1996), we launched a molecular approach aimed at characterizing the determinant of AG resistance in this species. A genomic library of *M. fortuitum* was transformed in *M. smegmatis*, and characterization of AG resistant clones allowed the identification of a *M. fortuitum* gene showing sequence similarity to *aac(2′)-Ia* of *P. stuartii*. The enzyme of *M. fortuitum* was named AAC(2′)-Ib (Ainsa et al., 1996) and was capable of acetylating gentamicin, but not kanamycin A, hence indicating that another AAC enzyme should be present in *M. fortuitum*. Similarly to *P. stuartii*, the *aac(2′)-Ib* gene was found in all strains of *M. fortuitum* regardless of the phenotype of AG resistance, suggesting other roles for the AAC(2′)-Ib in this species. Further studies (done by database searching or by southern blot analysis using the probe of *aac(2′)-Ib* gene) demonstrated the presence of *aac(2′)-I* genes in other mycobacterial species, including *M. tuberculosis* (the major pathogenic species in this genus), *Mycobacterium leprae*, and *M. smegmatis*, indicating thus that the presence of *aac(2′)-I* genes in mycobacteria could be universal (Ainsa et al., 1997). Interestingly, the expression of the *aac(2′)-Id* gene in *M. smegmatis* was driven from two promoters, and the strongest one produced a leaderless transcript having a GTG translation start codon at its 5′ end (Mick et al., 2008). Leaderless transcripts are those in which the transcription start site coincides with the translation start codon; although representing a rather unusual feature in the model organism *E. coli*, they are quite common in mycobacteria, where 25% of all transcripts are predicted to be leaderless (Shell et al., 2015); in fact, *eis* gene of *M. tuberculosis* (see the section “The Eis Protein Becomes a Novel Aminoglycoside Acetyltransferase,” below) is transcribed also as a leaderless mRNA (Zaunbrecher et al., 2009). In leaderless mRNAs, there is no Shine-Dalgarno sequence, and 70S ribosomes bind directly to the 5′-end of the mRNA in order to initiate translation (Shell et al., 2015).

### New Roles for AAC(2′) in Mycobacteria

Two major evidences supported that in mycobacteria, AAC(2′)-I enzymes could have additional roles other than acetylation of AGs containing 2′-amino group: first, the capability of amino sugars and diverse acyl-CoA molecules to inhibit the acetylating activity of mycobacterial crude extracts (Udou et al., 1989); and second, the implication of AAC(2′)-Ia (an enzyme of the same class) in acetylation of cell wall substrates in *P. stuartii*. In a series of laboratory mutants of *P. stuartii* expressing *aac(2′)-Ia* gene at different levels, it was found that the extent of peptidoglycan acetylation correlated with the activity of AAC(2′)-Ia, hence suggesting a partial contribution of this enzyme (along with other enzymes) to peptidoglycan acetylation. Many bacterial pathogens acetylate their peptidoglycan as a way to resist the action of muramidase enzymes. Under *in vitro* conditions, AAC(2′)-Ia was able to acetylate tobramycin having acetylated peptidoglycan as donor of acetyl groups (Payie et al., 1995, 1996; Payie and Clarke, 1997).

We investigated the hypothesis of mycobacterial AAC(2′)-I enzymes having also a role in cell wall metabolism, and a gene knock-out mutant of *M. smegmatis* deleted in *aac(2′)-Id* gene was constructed. This mutant (named EP-10) defective in AAC(2′)-I activity was more susceptible to gentamicin, tobramycin, dibekacin, and netilmicin than the parental wild-type strain, and crude extracts of *M. smegmatis* EP-10 failed to acetylate 2′-amino group containing AGs, whereas wild-type strains of *M. smegmatis* readily acetylated such AGs (Ainsa et al., 1997; Maurer et al., 2015). *M. smegmatis* EP-10 was also twofold more susceptible to lysozyme, a feature that has been associated with the extent of peptidoglycan acetylation (Ainsa et al., 1997). Hence, we concluded that in *M. smegmatis*, AAC(2′)-Id enzyme could also contribute to acetylation of peptidoglycan, since a less extensively acetylated peptidoglycan in the knock-out strain would be more susceptible to lysozyme degradation affecting cell viability.

### Biochemical Analysis of Mycobacterial AAC(2′)-I Enzymes

The presence of an AG (2′)-*N*-acetyltransferase gene in the genome of *M. tuberculosis* was intriguing. In this species, AAC activity had never been reported (Mitsuhashi et al., 1977) and the expression of the *aac(2′)-Ic* gene [annotated as Rv1258c gene in the *M. tuberculosis* H37Rv genome (Cole et al., 1998)] in the surrogate host *M. smegmatis* could not be associated with any change in the levels of susceptibility to AGs (Ainsa et al., 1997). We constructed a knock-out mutant of *M. tuberculosis* H37Rv deleted in the *aac(2′)-Ic* gene and observed that the mutant (named *M. tuberculosis* B1) was twofold more susceptible than the original wild-type strain to AGs containing a 2′-amino group such as gentamicin, tobramycin and dibekacin, and fourfold more susceptible to 6′-*N*-ethyl-netilmicin. This indicated that the *aac(2′)-Ic* gene was being expressed in *M. tuberculosis* although at a very low level, and that the AAC(2′)-Ic enzyme in *M. tuberculosis* would acetylate all these four AGs in the wild type strain; hence, acetylated AGs would bind less efficiently to the ribosome, and the AAC(2′)-Ic enzyme would contribute to basal AG resistance in this species.

In order to find the physiological role of this enzyme in *M. tuberculosis*, recombinant *E. coli*-produced AAC(2′)-Ic enzyme from *M. tuberculosis* was studied and found to efficiently acetylate AG antibiotics containing an amino group at the 2′ position (as expected; for example, *K*_m_ for kanamycin B was 1.4 μM), and surprisingly, it was also capable of acetylating (although to a much lesser extent) other AGs such as kanamycin A (*K*_m_ 320 μM) and amikacin (*K*_m_ 968 μM) that have a hydroxyl group in the 2′ position, hence suggesting this enzyme to be capable of both *N*- and *O*-acetylation (Hegde et al., 2001; Draker et al., 2003). This residual activity of AAC(2′)-Ic against kanamycin A and amikacin does not affect their bactericidal activity, and these two antibiotics are used as second line drugs against drug resistant *M. tuberculosis* (World Health Organization [WHO], 2010). The activity of *M. tuberculosis* AAC(2′)-Ic is dependent on metal ions, being inhibited by Cu^2+^ and Au^3+^ (Li et al., 2015).

Another study consisted in binding covalently the AGs kanamycin A, tobramycin, neamine and neomycin B to an agarose matrix in order to quantify the extent of AG acetylation by AAC enzymes and their subsequent ability to bind an artificial probe mimicking the A-site of the ribosome (Barrett et al., 2008). In such experimental model system, the AGs acetylated by *M. tuberculosis* AAC(2′)-Ic enzyme (only tobramycin, neamine and neomycin B) maintained their binding affinity with the probe mimicking A-site of the ribosome at detectable levels, maybe because the percent of AG acetylation by AAC(2′)-Ic was low. In contrast, these AGs were very efficiently acetylated by *E. coli* AAC(3) (at a different amino group) and had readily lost their affinity for binding this artificial probe. These experiments suggested that subtle differences in the structure of modified AGs (i.e., acetylation at the amino group in 2′ or 3 position) are sufficient to drastically affect their capability of binding to the A-site probe, and this would be expected to correlate with their activity as ribosome inhibitors. In consequence, in mycobacteria, AAC(2′)-I enzymes would not play a major role in resistance to these drugs (Barrett et al., 2008). High resolution crystal structures of AAC(2′)-Ic complexed with AGs demonstrated that this enzyme is a member of the GNC5 acetyltransferase superfamily and suggested a role in the synthesis of mycothiol, a metabolite that has a key role in regulating redox potential in mycobacteria (Vetting et al., 2002, 2003).

In agreement with the preferential *N*-acetylating activity over the *O*-acetylating activity that was found in the *M. tuberculosis* enzyme (Hegde et al., 2001; Draker et al., 2003), *M. abscessus* clinical isolates were found to be more susceptible to AGs containing a hydroxyl group at the 2′ position (such as amikacin and kanamycin A) than to AGs with a 2′-amino group (such as tobramycin, dibekacin and kanamycin B), as the latter group would be substrates of *M. abscessus* AAC(2′)-I enzyme (Maurer et al., 2015). In fact, crude extracts of *M. abscessus* efficiently acetylated kanamycin B, whereas kanamycin A was not acetylated at detectable levels. In this species, deletion of the *aac(2′)-I* gene resulted in increased susceptibility to kanamycin B, tobramycin, dibekacin and gentamicin C (all of them containing a 2′-amino group) (Rominski et al., 2017). These two reports demonstrate that in *M. abscessus*, the presence of an AAC(2′)-I enzyme contributes to decreased innate susceptibility to AGs containing a 2′-amino group (Luthra et al., 2018).

### Becoming a Drug Target: Developing Inhibitors of AAC(2′)-I

The interest in developing inhibitors against AAC(2′)-I enzymes came from a study in the non-tuberculous species *M. abscessus* (Maurer et al., 2014). It was found that AGs such as amikacin, gentamicin or tobramycin, which are normally bactericidal against *E. coli*, do not have such activity against *M. abscessus* or *M. smegmatis*. However, disruption of the chromosomally encoded *aac(2′)-I* gene in these species restored the bactericidal activity of these AGs (Maurer et al., 2014). Given that AGs are used as second-line drugs in treatment of multidrug resistant (MDR) tuberculosis infections, and also in the treatment of other infections caused by non-tuberculous mycobacteria, the possibility of developing compounds that could enhance bactericidal activity of current antimycobacterial treatments became an interesting approach.

To date, the only putative inhibitor of *M. tuberculosis* AAC(2′)-Ic is andrographolide, a natural product that was identified in methanolic extracts of a plant that were capable of inhibiting growth of *M. tuberculosis* strains (Prabu et al., 2015). *In silico* analysis predicted that this compound could potentially bind with a high affinity the AAC(2′)-Ic enzyme, as well as isocitrate lyase (a metabolic enzyme of the glyoxylate shunt, involved in persistence and virulence of *M. tuberculosis*) and other *M. tuberculosis* proteins (Prabu et al., 2015). However, the specificity of this binding and the ability to really inhibit such putative target proteins were not tested. Given that the gene encoding AAC(2′)-Ic is not essential in *M. tuberculosis*, a direct link between AAC(2′)-Ic inhibition and bacterial growth inhibition could be discarded. Hence, the ability of plant extracts containing andrographolide to inhibit growth of *M. tuberculosis* could be due to the presence of additional compounds in the extract, or to multiple effects on *M. tuberculosis* cells. Other *in silico* analysis revealed that AAC(2′)-Ic enzyme from *M. tuberculosis* could interact with ten other proteins (including a protein of a putative RND-like efflux pump), suggesting that inhibition of AAC(2′)-Ic could also impact many other metabolic processes, hence conferring this enzyme with a relevant role in drug discovery of antituberculosis agents (Joshi et al., 2013).

## The Eis Protein Becomes a Novel Aminoglycoside Acetyltransferase

Investigation of *M. tuberculosis* virulence factors lead to the identification of a protein, that was required for infecting and survival in human macrophages; this protein was named Eis for enhanced intracellular survival (Box 2) (Wei et al., 2000). Bioinformatic analysis revealed that Eis protein of *M. tuberculosis* was an acetyltransferase of the GCN-5 family (Samuel et al., 2007).

**Box 2.** Discovery and characterization of enhanced intracellular survival (Eis) protein of *M. tuberculosis.*

A genome wide investigation of the ability of *M. tuberculosis* for infecting macrophages resulted in the identification of a coding sequence that, once cloned in the non-pathogenic *M. smegmatis* species, conferred capacity for infecting the human macrophage-like cell line U937. This gene was named *eis* (Rv2416c in the *M. tuberculosis* genome; Cole et al., 1998) for enhanced intracellular survival (Wei et al., 2000), and was detected only in pathogenic species of mycobacteria. The promoter of the *eis* gene is similar to consensus *E. coli* sigma-70 dependent promoters (Roberts et al., 2004) and it is recognized by *M. tuberculosis* SigA sigma factor (Wu et al., 2009). The Eis protein was found to be mostly hydrophilic, but having a hydrophobic N-terminal end, so that it could be found in the cytosolic but also in other cell fractions such as the membrane, cell wall or among the secreted proteins of *M. tuberculosis* (Dahl et al., 2001). In fact, antibodies against Eis could detect this protein in the sera of tuberculosis patients (Dahl et al., 2001) and in the cytoplasm of infected macrophages (Samuel et al., 2007). Also, it was found that Eis protein, directly added to cultures of human monocytes, modulated the secretion of pro-inflammatory cytokines in a similar way to that found in *M. tuberculosis* infected cells (Samuel et al., 2007), hence suggesting a role of Eis as an effector protein. Further studies demonstrated that Eis inhibits the extra-cellular signal-regulated kinase 1/2 (ERK1/2) and JAK pathways, and in consequence it inhibited the production of TNF-alpha and IL-4, and stimulated the production of IFN-gamma and IL-10 (Lella and Sharma, 2007). The effect of Eis in increasing production of IL-10 was found to be related to Eis-mediated acetylation of histone H3, which binds the promoter of the human IL-10 gene (Duan et al., 2016). Hence, by disturbing cross-regulation of T-cells and impairing TH1 and TH2 response, Eis could mediate *M. tuberculosis* pathogenicity (Lella and Sharma, 2007). In fact, an isolate of the Beijing family (which are more transmissible and virulent than other *M. tuberculosis* genetic lineages (Hanekom et al., 2011) was found to contain elevated levels of Eis protein, mediated by increased expression of SigA (Wu et al., 2009). Other key factors in host immune response to tuberculosis are also mediated by Eis, which increased production of ROS and consequently modulated processes such as autophagy, inflammation, and cell death (Shin et al., 2010). These processes are started by Eis-dependent acetylation of dual-specificity phosphatase-16 (DUSP16)-mitogen-activated protein kinase phosphatase-7 (MKP-7), which dephosphorylates the JNK protein leading to its inactivation (Kim et al., 2012; Yoon et al., 2013). Other studies have revealed the activity of Eis for acetylating arylalkylamines such as histamine, octopamine, or tyramine, suggesting novel roles for this protein in *M. tuberculosis* pathogenicity (Pan et al., 2018).

Later on, the analysis of kanamycin resistant *M. tuberculosis* laboratory and clinical strains revealed mutations in the -10 and -35 regions of the *eis* gene promoter, which resulted in increased levels of *eis* mRNA and Eis protein. These mutations were related to low-level resistance to kanamycin (MIC 25 μg/ml), but not to amikacin (MIC < 4 μg/ml), whereas 16S rRNA mutations confer higher levels of resistance to kanamycin (MIC > 80 μg/ml) and frequently cross-resistance to amikacin. These *eis* mutants also displayed increased levels of AAC activity, hence demonstrating that Eis was a novel class of AAC, highly divergent from all other previously known AACs. Eis is capable of acetylating kanamycin more efficiently than amikacin, and streptomycin was not found to be a substrate of Eis (Zaunbrecher et al., 2009). From then on, detection of mutations in *eis* promoter has become a relevant assay in clinical microbiology laboratories for determining susceptibility to kanamycin (Georghiou et al., 2012); the diagnostic and clinical implications of these tests are beyond the scope of this review.

Formation of stable hexamers by Eis is required for its AAC activity (Ganaie et al., 2011; Anand and Sharma, 2018), which can acetylate multiple amine groups of different AG antibiotics, including netilmicin, sisomicin, neamine, ribostamycin, paromomycin, neomycin B, kanamycin, amikacin, tobramycin and hygromycin, resulting in mono-, di-, tri, and tetraacetylated products (Chen et al., 2011; Houghton et al., 2013a), being able to use not only acetyl-CoA but also other acyl-CoA derivatives (Chen et al., 2012a). The Eis protein works by a random-sequential bisubstrate mechanism of acetylation (Tsodikov et al., 2014). Interestingly, Eis is also able to acetylate capreomycin, a polypeptide second-line antituberculosis drug commonly used in the treatment of MDR tuberculosis infections (Reeves et al., 2013). Several metal ions such as Au^3+^, Cd^2+^ and Zn^2+^ inhibited Eis activity *in vitro* (Li et al., 2015).

Other mycobacterial species such as *M. smegmatis* and *M. abscessus* have ortholog (and even paralog) *eis* genes, although the Eis proteins have distinct biochemical features and impact on AG susceptibility in comparison with Eis of *M. tuberculosis* (Chen et al., 2012b; Rominski et al., 2017). For example, *M. abscessus* has two *eis* genes, and the deletion of one (but not the other one) resulted in altered AG susceptibility (Rominski et al., 2017; Luthra et al., 2018). Consistently with these findings, mutational changes in the amino acid residues lining the substrate binding site of *M. tuberculosis* Eis altered its substrate specificity (Jennings et al., 2013).

It is important to note that in *M. tuberculosis*, transcription of the *eis* gene is activated by the regulator WhiB7 (Reeves et al., 2013). Mutations in the promoter of *whiB7* gene that led to increase in the mRNA of this gene resulted in increased expression of *eis* gene, along with other genes such as *rv1258c* (encoding the Tap efflux pump; Ainsa et al., 1998), hence resulting in cross resistance to several drugs including kanamycin (mediated by Eis protein) or streptomycin (mediated by Tap efflux pump). Similarly, in *M. abscessus*, WhiB7 controlled the expression of one of the two *eis* genes in this species and also that of the *erm(41)* gene, which encodes a ribosomal methyltransferase that by altering target structure is associated with resistance to macrolide antibiotics (Pryjma et al., 2017; Luthra et al., 2018). Subinhibitory concentrations of clarithromycin induced the *whiB7* gene and consequently decreased Eis-mediated susceptibility to AGs, such as amikacin that is currently used in the treatment of *M. abscessus* infections (Pryjma et al., 2017).

### Aminoglycosides and Beyond…

The unusual properties of Eis acetyltransferase include its capability for acetylating peptides and proteins (Kim et al., 2012; Houghton et al., 2013b; Yoon et al., 2013), in contrast with other AACs. The *M. tuberculosis* nucleoid-associated protein HU (encoded by Rv2986c gene) can be acetylated by Eis on multiple lysine residues, hence decreasing its ability to interact with DNA, and altering its DNA compactation activity (Ghosh et al., 2016; Green et al., 2018). Overexpression of Eis led to a hyperacetylation of HU protein, and consequently, to a decompactation of the genome (Ghosh et al., 2016). The reverse effect (condensation of relaxed DNA) could be reached through the deacetylation of HU protein, which is mediated by a Sir2 family protein from *M. tuberculosis* encoded by the Rv1155c gene (Anand et al., 2017; Green et al., 2018). Controlling the architecture of DNA is a key process in any bacteria, and so, the HU protein is essential for *M. tuberculosis*. In fact, inhibitors of HU have been discovered (Bhowmick et al., 2014), which could act in synergy with potential inhibitors of Eis, as described in the next section.

### Finding Inhibitors of Eis Protein

The crystal structure of *M. tuberculosis* Eis protein was determined by several groups (Chen et al., 2011; Kim et al., 2012), which has been useful for determining docking properties of potential inhibitory compounds; also, its comparison with the crystal structure of *M. smegmatis* Eis protein revealed several distinct structural features that may account for the biochemical and substrate differences between the two proteins (Kim et al., 2014). A first screening of potential inhibitors of Eis from *M. tuberculosis* resulted in the identification of 25 molecules (including the antiseptic chlorhexidine) that inhibited Eis with IC_50_ values in the low micromolar range. In addition, this inhibition was specific to the *M. tuberculosis* Eis protein, since these molecules could not inhibit significantly AACs from AAC(2′), AAC(3), and AAC(6′) families (Green et al., 2012), nor Eis protein from *Bacillus anthracis* (Green et al., 2015a). Later studies revealed the presence of *eis*-like genes in many pathogenic and non-pathogenic bacteria (remarkably, many mycobacterial species have two or even three paralogs of the *eis* gene), and chlorhexidine was capable of inhibiting (to different levels) all Eis proteins that were capable of acetylating AGs (Green et al., 2015b).

A second screening of a larger collection of small-molecule compounds resulted in the identification of several families of compounds capable of inhibiting Eis activity. These contained diverse chemical scaffolds (Garzan et al., 2016a,b, 2017; Willby et al., 2016; Ngo et al., 2018). Besides, these inhibitors were highly selective for Eis, and did not inhibited AACs from other families (Garzan et al., 2016b). More importantly, as these inhibitors bound in the AG pocket of the Eis protein, they were able to reverse kanamycin resistance of a *M. tuberculosis* isolate (Garzan et al., 2016b; Willby et al., 2016; Ngo et al., 2018). Some of these inhibitors, such as those based on a pyrrolo[1,5-a]pyrazine scaffold, also lacked any toxicity on mammalian cell lines (Garzan et al., 2017).

## Other Aminoglycoside-Modifying Enzymes in Mycobacteria

Genome-wide analysis of *M. tuberculosis* genome identified only one other potential AAC (encoded by the Rv1347c gene), although such enzymatic activity could not be detected on the recombinant protein (Draker et al., 2003). Later studies related the product of the Rv1347c gene with a role in the synthesis of mycobactin, the mycobacterial siderophore (Card et al., 2005).

Leaving apart AACs, only a few reports of other classes of aminoglycoside-modifying enzymes have been done in mycobacteria. An APH enzyme of the APH(3″) family, conferring resistance to the AG streptomycin only, has been characterized in *M. fortuitum* (Ramon-Garcia et al., 2006) and *M. abscessus* (Dal Molin et al., 2018; Luthra et al., 2018); the latter species encodes up to 11 additional putative APH enzymes (Ripoll et al., 2009). Furthermore, a putative APH of the APH(3′) class, encoded by the Rv3168 gene of *M. tuberculosis*, was identified and expressed as a recombinant enzyme in *E. coli*, being related to kanamycin phosphotransferase activity (Ahn and Kim, 2013).

## Back to the Start Point: Eis in *M. fortuitum*

We started this review referring to previous work that has shown that crude extracts of *M. fortuitum* harbored AAC activity having gentamicin, tobramycin, netilmicin and its derivatives 2′-*N*-ethyl- and 6′-*N*-ethyl-netilmicin, kanamycin A and kanamycin B as substrates. So far, the only AG acetyltransferase identified in this species has been AAC(2′)-Ib (Ainsa et al., 1996), which cannot explain the acetyltransferase activity against kanamycin A and 2′-*N*-ethyl-netilmicin detected in *M. fortuitum* crude extracts. Later studies characterized Eis AAC in diverse mycobacterial species, but no report were been done on a putative Eis protein in *M. fortuitum*. In view of the universal presence of Eis proteins in mycobacteria, and its activity as AAC, we hypothesized that *M. fortuitum* could also have a putative Eis protein that would be responsible for the acetyltransferase activity against kanamycin A and 2′-*N*-ethyl-netilmicin detected in crude extracts of this species, as it was reported earlier (Hull et al., 1984; Ainsa et al., 1996).

**FIGURE 1:**
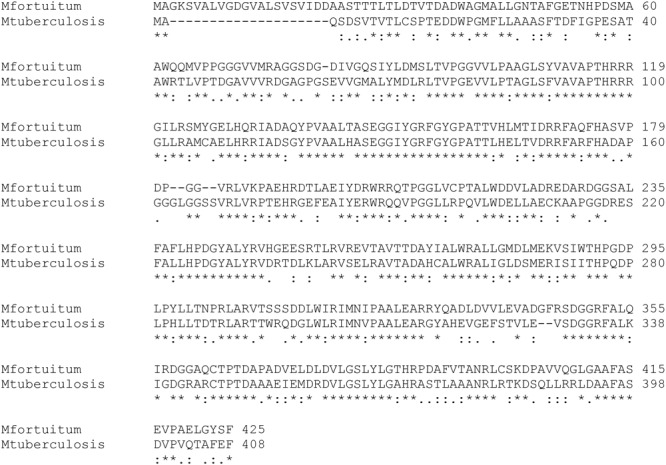
Comparison of the amino acid sequences of Eis proteins from *M. fortuitum* and *M. tuberculosis*. Symbols under the sequence alignment: Asterisks (^∗^) indicate positions with identical amino acid in both proteins. Colons (:) indicate positions which have amino acids with strongly similar properties. Periods (.) indicate positions which have amino acids with weakly similar properties.

Thus, we first ascertained the existence of an *eis* gene in the genome of *M. fortuitum* (Ho et al., 2012), which presented a 94% of identity with respect to the one reported in *M. tuberculosis;* the sequence of both Eis proteins from *M. tuberculosis* and *M. fortuitum* also presented high levels of identity (Figure 1). We designed two oligonucleotides for amplifying specifically a DNA fragment from *M. fortuitum* genome containing *eis* gene. This DNA fragment was subsequently cloned in pMV261 vector (Stover et al., 1991), which expresses genes constitutively under the control of the *hsp60* gene promoter, resulting in plasmid pFS2. Given that this plasmid still contains the Tn*903*-derived aminoglycoside-3′-phosphotransferase (*aph*) gene (present in the original pMV261 cloning vector) conferring kanamycin resistance as selection marker, we anticipated that *M. smegmatis* strains harboring pMV261 vector or its derivatives would be intrinsically resistant to kanamycin A; this would prevent from determining whether this antibiotic is a substrate of the *M. fortuitum* Eis protein. Additionally, the selection marker could have cross-effect with other AGs and thus interfering with the resistance phenotype conferred by *eis* gene. To circumvent these problems, we generated a derivative of pFS2 through the disruption of the *aph* gene with the ampicillin resistance cassette (*bla* gene) from pGEM^®^-T easy (Promega). The resistance to 2′-*N*-ethylnetilmicin conferred by *eis* gene, as demonstrated for the parental plasmid pFS2 (see Table 1) was used as resistance marker for transformant selection and plasmid maintenance. This process resulted in plasmid pEAC which contains the *M. fortuitum eis* gene and no other determinant of AG resistance.

**Table 1:** MICs (μg/ml) of antibiotics of different structural families in the *M. smegmatis* mc^2^ 155 strains that overexpress *eis* gene from *M. fortuitum*.

Strain	*M. smegmatis* mc^2^ 155			
Plasmid	None	pMV261	pFS2	pEAC
Marker gene on plasmid	None	*aph*	*aph*	*bla*
*M. fortuitum eis* gene	–	–	+	+
Gentamicin	0.78-1.56	0.78	3.12	3.12
2′-*N*-ethyl netilmicin	6.25	3.12–6.25	50–100	50
6′-*N*-ethyl netilmicin	3.12	3.12	3.12–6.25	25
Kanamycin A	1.56	>100	>100	7.8
Kanamycin B	6.25	>100	>100	12.5
Hygromycin	15.6	15.6	31.2–62.5	62.5
Amikacin	0.39	0.39	0.39	0.39
Capreomycin	1.95	1.95	3.9	3.9
Streptomycin	0.25	0.25	0.25	0.25
Spectinomycin	62.5	31.2–62.5	62.5	62.5

*The values separated by a dash indicate that growth was detected in both concentrations. The range of antibiotic concentrations spanned from 0.78 *μ*g/ml to 100 *μ*g/ml, and from 0.25 *μ*g/ml to 125 *μ*g/ml; a twofold difference in the MIC was not considered as significant. aph: aminoglycoside 3′-phosphotransferase from Tn903; bla: beta-lactamase.*

The three plasmids [original empty vector pMV261 as a control, and the two plasmids (pFS2 and pEAC) containing *M. fortuitum eis* gene] were introduced in *M. smegmatis* mc^2^ 155 in order to over-express the ortholog *eis* gene from *M. fortuitum* and to elucidate its hypothetical implication in AG susceptibility. The antibiotic susceptibility assay was made, based on a double dilution protocol with the addition of resazurin dye (Palomino et al., 2002).

We observed that plasmids pFS2 and pEAC produced detectable changes in AG susceptibility of *M. smegmatis* mc^2^ 155, which can be attributed to the expression of the plasmid-borne *M. fortuitum eis* gene. The major shift in the MICs was detected for 2′-*N*-ethylnetilmicin (Table 1), since the MIC increased from 3.12 to 6.25 μg/ml in the control strains to 50–100 μg/ml in the strains containing *M. fortuitum* Eis, accounting for a 8- to 32-fold increase; similar changes were observed for 6′-*N*-ethylnetilmicin (8-fold increase in the MIC). A moderate decrease in the susceptibility to kanamycin A (fivefold), hygromycin (twofold to fourfold) and gentamicin (twofold to fourfold) was also observed. Finally, slight changes (twofold) in the MICs were detected for kanamycin B and capreomycin; this finding is consistent with previous reports on the activity of *M. tuberculosis* Eis against capreomycin (Reeves et al., 2013). The levels of susceptibility to the AGs amikacin, streptomycin and spectinomycin, and to other non-AG compounds tested (isoniazid, rifampicin, ethambutol, ciprofloxacin, tetracycline, chloramphenicol) were not altered significantly by the presence of the plasmid-encoded *eis* gene (Table 1 and data not shown), suggesting that either these antimicrobials are not substrates of the Eis enzyme or the corresponding acetylations (if any) might just not affect antibacterial activity.

## Concluding Remarks

The presence and activity of AAC(2′)-I and Eis AACs in mycobacteria have clearly demonstrated that their primary role is little related with susceptibility to AGs.

In the case of AAC(2′)-I enzymes, its presence in phylogenetically distant genera as *Providencia* and *Mycobacterium* remains to be an evolutionary mystery. In contrast with this restricted distribution of AAC(2′)-I enzymes among bacteria, Eis enzymes seem to be more widely distributed, being present even in non-pathogenic and environmental species, which suggest a general function of Eis-like enzymes in bacterial metabolism, and virtually excludes any potential selection of *eis* genes due to the use of AGs, or its horizontal transfer from other species.

It is clear that in mycobacteria ribosomal modifications constitute the major mechanism of AG resistance, given that only one or two copies of ribosomal RNA operons are present in these species, hence making likely the acquisition of mutations conferring high levels of AG resistance. Then, AAC(2′)-I enzymes only contribute modestly to innate low level susceptibility to AGs, and despite other roles have been suggested in the literature for mycobacterial AAC(2′)-I enzymes, their relevance as potential drug targets is still modest, especially in comparison with Eis acetyltransferase. The contribution of Eis acetyltransferase to virulence of *M. tuberculosis*, and the finding that Eis is related with resistance to kanamycin (a second line drug for the treatment of tuberculosis) in clinical isolates has greatly attracted the attention and promoted the interest in developing Eis inhibitors. In some way, Eis inhibitors would fall into the class of anti-virulence and anti-resistance mechanisms compounds, which is a trending topic in the age of antimicrobial resistance. Globally, antimicrobial resistance is a major public health threat, and multi and extensively drug resistant (MDR, XDR) tuberculosis is a case of particular concern, so progress and major advances that can be expected from the coming years will be greatly welcomed.

## Author Contributions

JA, LR, and CM contributed to the conception and design of the study. FS-G, EA-C, AL, LR, and EP-H carried out the experimental work. FS-G, EA-C, and JA wrote the manuscript. All authors contributed to manuscript revision, read and approved the submitted version.

## Conflict of Interest Statement

The authors declare that the research was conducted in the absence of any commercial or financial relationships that could be construed as a potential conflict of interest.
